# Angiogenic and anti-inflammatory properties of micro-fragmented fat tissue and its derived mesenchymal stromal cells

**DOI:** 10.1186/s13221-016-0037-3

**Published:** 2016-08-18

**Authors:** Valentina Ceserani, Anna Ferri, Angiola Berenzi, Anna Benetti, Emilio Ciusani, Luisa Pascucci, Cinzia Bazzucchi, Valentina Coccè, Arianna Bonomi, Augusto Pessina, Erica Ghezzi, Offer Zeira, Piero Ceccarelli, Silvia Versari, Carlo Tremolada, Giulio Alessandri

**Affiliations:** 1 Cellular Neurobiology Laboratory, Department of Cerebrovascular Diseases, IRCCS Neurological Institute C. Besta, Via Celoria 11, 20131 Milan, Italy; 2 Department of Clinical and Experimental Sciences, Institute of Pathological Anatomy, University of Brescia, Brescia, Italy; 3 Laboratory of Clinical Pathology and Neurogenetic Medicine, Fondazione IRCCS Neurological Institute C. Besta, Milan, Italy; 4 Department of Veterinary Medicine, University of Perugia, Perugia, Italy; 5 Department of Biomedical, Surgical and Dental Sciences, University of Milan, Milan, Italy; 6 San Michele Veterinary Hospital, Tavezzano con Villavesco, Lodi, Italy; 7 Image Institute, Milan, Italy

## Abstract

**Background:**

Adipose-derived mesenchymal stromal cells (Ad-MSCs) are a promising tool for advanced cell-based therapies. They are routinely obtained enzymatically from fat lipoaspirate (LP) as SVF, and may undergo prolonged *ex vivo* expansion, with significant senescence and decline in multipotency. Besides, these techniques have complex regulatory issues, thus incurring in the compelling requirements of GMP guidelines. Hence, availability of a minimally manipulated, autologous adipose tissue would have remarkable biomedical and clinical relevance. For this reason, a new device, named Lipogems® (LG), has been developed. This ready-to-use adipose tissue cell derivate has been shown to have *in vivo* efficacy upon transplantation for ischemic and inflammatory diseases. To broaden our knowledge, we here investigated the angiogenic and anti-inflammatory properties of LG and its derived MSC (LG-MSCs) population.

**Methods:**

Human LG samples and their LG-MSCs were analyzed by immunohistochemistry for pericyte, endothelial and mesenchymal stromal cell marker expression. Angiogenesis was investigated testing the conditioned media (CM) of LG (LG-CM) and LG-MSCs (LG-MSCs-CM) on cultured endothelial cells (HUVECs), evaluating proliferation, cord formation, and the expression of the adhesion molecules (AM) VCAM-1 and ICAM-1. The macrophage cell line U937 was used to evaluate the anti-inflammatory properties, such as migration, adhesion on HUVECs, and release of RANTES and MCP-1.

**Results:**

Our results indicate that LG contained a very high number of mesenchymal cells expressing NG2 and CD146 (both pericyte markers) together with an abundant microvascular endothelial cell (mEC) population. Substantially, both LG-CM and LG-MSC-CM increased cord formation, inhibited endothelial ICAM-1 and VCAM-1 expression following TNFα stimulation, and slightly improved HUVEC proliferation. The addition of LG-CM and LG-MSC-CM strongly inhibited U937 migration upon stimulation with the chemokine MCP-1, reduced their adhesion on HUVECs and significantly suppressed the release of RANTES and MCP-1.

**Conclusions:**

Our data indicate that LG micro-fragmented adipose tissue retains either *per se*, or in its embedded MSCs content, the capacity to induce vascular stabilization and to inhibit several macrophage functions involved in inflammation.

## Background

Mesenchymal stromal cells (MSCs) are multipotent cells present in the stroma of many (if not all) human organs and tissues. They possess multi-lineage differentiation capacity [31, 32 ] and a potent paracrine activity [8, 30], which makes them an attractive tool for cell-based therapies [8, 28].

Among most of the MSC sources, adipose tissue is ideal because of the abundance, easy access and the simple isolation procedures. Indeed, adipose-derived MSCs (Ad-MSCs) can be easily obtained by a minimal invasive surgical procedure and expanded *in vitro*. In addition, Ad-MSCs have been shown to possess strong regenerative properties when transplanted *in vivo* in experimental animal models [11, 24, 33].

However, as for other MSC types, the use of Ad-MSCs is limited because of the high number of cells required in most of the clinical protocols in current human trials [17, 42]. Ad-MSCs are routinely obtained enzymatically from fat lipoaspirates (LP) as SVF and may undergo prolonged *ex vivo* expansion with significant increased risk of cell transformation and senescence [41], which leads to clinical results below the expectations. Besides, these techniques have complex regulatory issues [36].

For this reason, it is necessary to develop new procedures to obtain Ad-MSCs with minimal manipulation techniques. To this purpose, prof. Tremolada and colleagues [4] recently developed an enzyme free technology able to obtain a micro-fragmented fat preparation named Lipogems® (LG) containing a significant number of Ad-MSCs. This novel technology reduces the size of the adipose tissue clusters by means of mild mechanical forces while eliminating pro-inflammatory oil and blood residue. The technique is gentle and intra-operatively provides micro-fragmented fat in a short time (15-20’), without expansion and/or enzymatic treatment. This non-expanded micro-fragmented adipose tissue has been shown to possess regenerative properties, particularly when injected into inflammatory or ischemic tissues [5]. In the present study, we aimed to establish if LG placed in culture in a serum free medium retained efficient cells able to release potential active molecules. In particular, we investigated the properties of LG and its derived MSCs (LG-MSCs) population, focusing on their capacity to affect angiogenesis and inflammation through a paracrine activity.

## Methods

### Lipogems processing from adipose tissue

According to the policies approved by the Institutional Review Boards for Human Studies local ethical committees (IRB 48/2013, Istituto Neurologico Carlo Besta), all tissue samples were obtained after signed informed consent (2 male and 3 female, age ranging from 34–64). Lipogems (LG) was prepared as previously described [4]. Briefly, around 100 ml of adipose tissue was processed using LG device (provided free of charge for this study by Lipogems® International Spa). The harvested fat was then introduced into the Lipogems® processing kit. The Lipogems® system consists of a disposable kit for the aspiration, processing and reinjection of autologous adipose tissue. Its core is a disposable and closed device filled with saline solution that progressively reduces the size of adipose tissue clusters by means of mild mechanical forces and eliminates pro-inflammatory oil and blood residues through a minimal manipulation “enzyme free” technique in a closed and aseptic system completely prefilled by room temperature physiological solution. The technique is gentle and intra-operatively provides micro-fragmented fat in a short time (15-20’), without expansion and/or enzymatic treatment.

### Isolation, expansion and characterization of LG-MSCs

Human LG specimens (about 2 ml for each sample) were processed for MSC isolation. The LG-MSCs were isolated following a similar procedure described for fat specimens [11, 41]. Briefly, sterile LG samples were repeatedly washed with PBS (Phosphate buffered saline) for blood residual by centrifugation at 250 x g. After liquid phase removal, collagenase solution (0,25 % w/v) (SIGMA St. Louis, Mo, USA) plus 200 μl DNAse (Sigma Aldrich, St. Louis, MO, USA) at 1:100 dilution were added to LG. All tubes were then incubated overnight at 37 °C. After enzymatic digestion, cells were washed by centrifugation at 250 x g for 10’. Pellets were re-suspended in IMDM (Iscove’s Modified Dulbecco’s Medium) added with 5 % FBS (Fetal Bovine Serum) (Gibco, Life Technologies, Monza, Italy), seeded into T25 culture flasks, and incubated at 37 °C in a humidified atmosphere containing 5 % CO_2_. Alternatively, an aliquot of the cell pellet was seeded on chamber slide, and cultured for 72 h before processing for immunocytochemistry. The following day, the medium was aspirated and replaced with IMDM + 5 % FBS and 50 ng/ml bFGF (LONZA Walkersville, MD. USA). Upon 5–7 days of culture, the adherent LG-derived cells were harvested with trypsin and processed for the removal of CD31^+^ cells by using magnetic beads (Invitrogen, Italy, CELLection™ Pan Mouse IgG Kit,) as previously described [3]. Briefly, cells were counted and re-suspended in PBS+ 0.2 % BSA at a concentration of 10^6^/ml. Around 5x10^6^ CD31^+^ pan beads were added to cells and incubated at 4 °C for 30’ under gentle rotation. At the end of incubation, the CD31^+^ cells (i.e. the ECs) bound to magnetic beads were separated with a magnetic apparatus, while the remaining CD31^−^ cells, i.e. LG-MSCs, were expanded in IMDM +5 % FBS and routinely passed at 70–80 % confluence. For this study, cultures were not expanded for more than 3–5 *in vitro* passages.

Flow cytometry (FC) was used to confirm LG-MSC mesenchymal phenotype at passages 3. Briefly, after trypsinization, cells were re-suspended in PBS at a concentration of 1x10^5^ /100 μl and incubated with 10 μl of conjugated primary antibody for 30’ at 4 °C in the dark. Phycoerythrin (PE) conjugate-antibodies were used: anti-human CD90PE (Millipore, Billerica, MA, USA; working dilution 1:10), anti-human CD105PE, anti-human CD73PE, anti-human CD44PE (BD Pharmingen™, San Jose, CA, USA; working dilution 1:10). Aspecific staining was determined with appropriate Isotype Control. At least 20,000 events were acquired for each sample on a FACS Advantage SE (BD Bioscience, San Diego, CA, USA) flow cytometer and the acquisition analyses were performed using a CellQuest software (BD Bioscience, San Diego, CA, USA).

### Preparation of conditioned medium (CM) from LG (LG-CM) and LG-MSCs (LG-MSC-CM)

Two different procedures for preparation of LG-CM and LG-MSCs-CM have been used because of the consistent secretions of growth factors and cytokines for Luminex analysis. Briefly, freshly LG was washed in PBS at least three time by centrifugation at 250 g for 10’. LG was then aspirated and seeded (around 2 ml) in T25 flask in 5 ml of IMDM serum free medium (a similar amount of LG was used for cells extraction with collagenase). The flasks (five for each LG preparation) were incubated for 5 days at 37 °C in 5 % CO2. At the end of the incubation, medium was aspirated and centrifuged at 250 x g for 10’. LG-CM was filtered 0.22 μ, aliquoted and stored at −20 °C until use. LG-MSCs-CM was prepared upon harvesting LG-MSCs from culture at passages 3 starting from CD31^+^ selection that eliminate ECs from primary culture. Around 2x105 LG-MSCs were seeded in 5 ml of IMDM + 5 % FCS and incubated at 37 °C for 3 days. At the end of the incubation, LG-MSCs-CM was centrifuged, filtered, and stored at −20 °C until used.

### Histology and Immunohistochemistry

LG and LP samples from 5 patients were fixed in 10 % formalin (Bio-Optica, Milan, Italy), embedded in paraffin (Bio-Optica, Milan, Italy) and then processed for histology and immunohistochemistry. Hematoxylin and eosin (Bio-Optica, Milan, Italy) stained serial sections of five micrometer thickness were immunostained for the detection of proteins of interest according to standard protocols [15]. Briefly, the sections were transferred to glass slides coated with poly-lysine, deparaffinized in 100 % xylene, and rehydrated in graded ethanol. All samples were then processed by the avidine-biotin peroxidase complex method according to manufacturer’s recommendations (DakoCytomation, Carpenteria CA, USA, LASB kit). The antibodies used for immunohistochemistry on LP and LG and immunocytochemistry on cells isolated from LG enzymatic digestion and cultured for 40–72 h and from LG-derived MSCs were: anti-NG2 (SANTA CRUZ, Biotechnology, California USA, working dilution 1:50), anti-CD146 (LEICA Biosystem Nussloch Germany, working dilution 1:50), anti-smooth muscle actin (SMA) (BIO-OPTICA Milan, Italy, working dilution 1:1000), anti-CD31 (LEICA Biosystem Nussloch 2015 Germany, working dilution 1:50), anti- vascular endothelial growth factor (VEGF) (BIO-OPTICA, Milan, Italy, working dilution 1:100), anti-CD105 (MONOSAN, Uden The Netherlands, working dilution 1:50), anti-CD34 (LEICA, Biosystem Nussloch Germany, working dilution 1:200), anti-CD44 (DAKO Glostrup, Denmark, working dilution 1:50), anti-transforming growth factor β1 (TGFβ1) (SANTA CRUZ Biotecnology, California, USA, working dilution 1:200).

### Evaluation of the angiogenic potential of LG and LG-MSCs

To assess the angiogenic potential of LG and LG-MSCs, we investigated the activity of their conditioned medium (CM) on Human Umbilical Vein Endothelial Cells (HUVECs) (LONZA Walkersville, MD. USA). HUVECs were routinely maintained in EGM bullet kit (LONZA Walkersville, MD. USA) plus 10 % FBS. HUVEC proliferation assay was performed as previously described [1]. Briefly, HUVECs were harvested from culture flasks by trypsin. After enzyme inactivation and centrifugation, cells were re-suspended in EGM basal medium + 0.2 % BSA and counted. To evaluate the growth response to LG-CM and LG-MSCs-CM, 5x10^4^/ml HUVECs were seeded into a 24-multiwell plate (Corning, NY, USA) coated with collagen type I. After adhesion, medium was aspirated and replaced with EGM complete medium + 10 % FBS supplemented or not with different dilutions of LG-CM and LG-MSCs-CM. Positive control growth medium consisted in EGM Bullet kit complete medium. After 72 h, cells were washed, fixed and stained. Cells were counted in triplicate with a calibrated ocular eyepiece in 10 different fields (40x magnification). To test the effect of LG-CM and LG-MSCs-CM on HUVEC tube formation, we used growth factor reduced-matrigel assay (Sigma) as described by Kleinman et al. [19]. Briefly, around 50 μl of matrigel was placed into cold (4 °C) 96-multiwell plate (Corning, NY, USA) at 37 °C for 30’ until jellification. HUVECs were then seeded on matrigel at a concentration of 10^4^ cells/well in 50 μl of EGM basal medium diluted (from 1:1 to 1:8) or not with LG-CM and LG-MSCs-CM. At day 1 and 5, the number of HUVEC tube formations were counted with an inverted microscope (10x magnification) and reported as n° of tube structures/field. To evaluate the content of angiogenic factors in both LG-CM and LG-MSCs-CM, Luminex analysis was performed (Labospace, Milan, Italy). Samples were analyzed according to the manufacturer’s protocol. AM expression on HUVECs was analysed by flow cytometry (FC) after exposure to LG-CM and LG-MSCs-CM at various dilutions (from 1:1 to 1:8). Briefly, HUVECs were washed and incubated for 24 h with LG-CM or LG-MSCs-CM. Cells were then washed, trypsinized, re-supsended in PBS, and processed for FC analysis as described above. Anti-CD106/VCAM-1-phycoerythrin (Serotech, Italy) and anti-CD54/ICAM-1- fluorescein isothiocynate (Immunotech, Milan, Italy) antibodies were used.

### Evaluation of anti-inflammatory activity of LG and LG-MSCs

The anti-inflammatory activity of LG and LG-MSCs was tested by treating the U937 monocyte cell line (ATCC Manassas VA, USA) with LG-CM and LG-MSCs-CM. Cells were routinely maintained in RPMI + 10 % FBS and passed twice a week. Corning Costar Transwell 5 μm pore size supports (Celbio, Milan, Italy) were used to test the effects of LG-CM and LG-MSCs-CM on U937 migration. MCP-1 chemokine (Sigma Aldrich, St. Louis, MO, USA) was used as positive chemotactic factor. For each test, 10^6^ cells in 200 μl of IMDM +0.2 % BSA were routinely placed at the top of the membrane insert (the upper compartment of the well). To evaluate spontaneous migration, 500 μl of control IMDM + 0.2 % BSA medium were added to the lower compartment of the wells. To evaluate LG-CM and LG-MSCs-CM activity, different dilutions (from 1:1 to 1:8) were added in the lower compartment of each well in the presence or in the absence of MCP-1 (10 ng/ml). Migration assay was carried out for 6 h at 37 °C in 5 % CO_2_ [3]. The membrane inserts were then removed, fixed in 10 % formalin and stained with Wright’s solution (Sigma Aldrich, St. Louis, MO, USA). Cells attached to the upper surface of the filter were removed with a swab and cells migrated across the membrane were counted by examining the lower surface with a microscope. Data are reported as the total number of cells found in 10 different fields for each membrane at 40x magnification. Each determination was performed in duplicate. Since interaction of inflammatory cells to endothelium is an important step in inflammation [29], adhesion of U937 to HUVECs was performed following a previously described procedure [31]. Briefly, HUVECs were seeded onto collagen-coated 24-multiwell plates (Corning, NY, USA) at a concentration of 5x10^4^/500 μl in complete endothelial basal medium EGM Bullet kit. The plates were incubated for 4 to 5 days to obtain a cell monolayer. HUVECs were activated by adding tumor necrosis factor-*alpha* (TNFα; 25 ng/ml; Sigma Aldrich) for 12 h. HUVECs were then washed with PBS and allowed to interact with U937 (10^5^ cells/well) for 30’ in RPMI-1640 (EuroClone Milano, Italy) containing 0.2 % BSA (control). Alternatively, U937 were primed for 12 h with different dilutions (from 1:1 to 1:8) of LG-CM and LG-MSCs-CM and then seeded on HUVECs (test conditions). The unbound U937 were removed by three washes with warm PBS and U937 attached to HUVECs were fixed for 5’ with 100 μl of cold methanol and stained with Diff Quick (Merz-Dade, Dudingen, Switzerland) for 30’ at room temperature. The plates were then washed several times with deionized water and the U937 bound to HUVECs were counted with a calibrated eyepiece in 15 different fields (40× magnification). Each test was performed in quadruplicate. ELISA-kits were used to quantify the production of RANTES and MCP-1 (R&D Systems, UK) by U937 under basal culture conditions, in the presence of Lipopolysaccharide (LPS) inflammatory stimuli (1ug/ml, Sigma) combined or not with different dilutions of LG-MSCs-CM and LG-CM. All the data were normalized for 10^6^ cells in 24 h incubation subtracting the basal level of the same chemokines present in the LG-MSCs-CM and in LG-CM used.

#### Statistical analysis

Statistical analysis was performed with the Statistical Package for Social Science (SPSS version 13, IBM, Armonk, NY, USA). Statistical differences were evaluated by the analysis of variance followed by Tukey-Kramer multiple comparison test and by the two-tailed, unpaired Student’s *t*-test. *P* ≤ 0.05 was considered statistically significant as indicated in the figures.

## Results

### LG and LG-MSC characterization

**Fig. 1:**
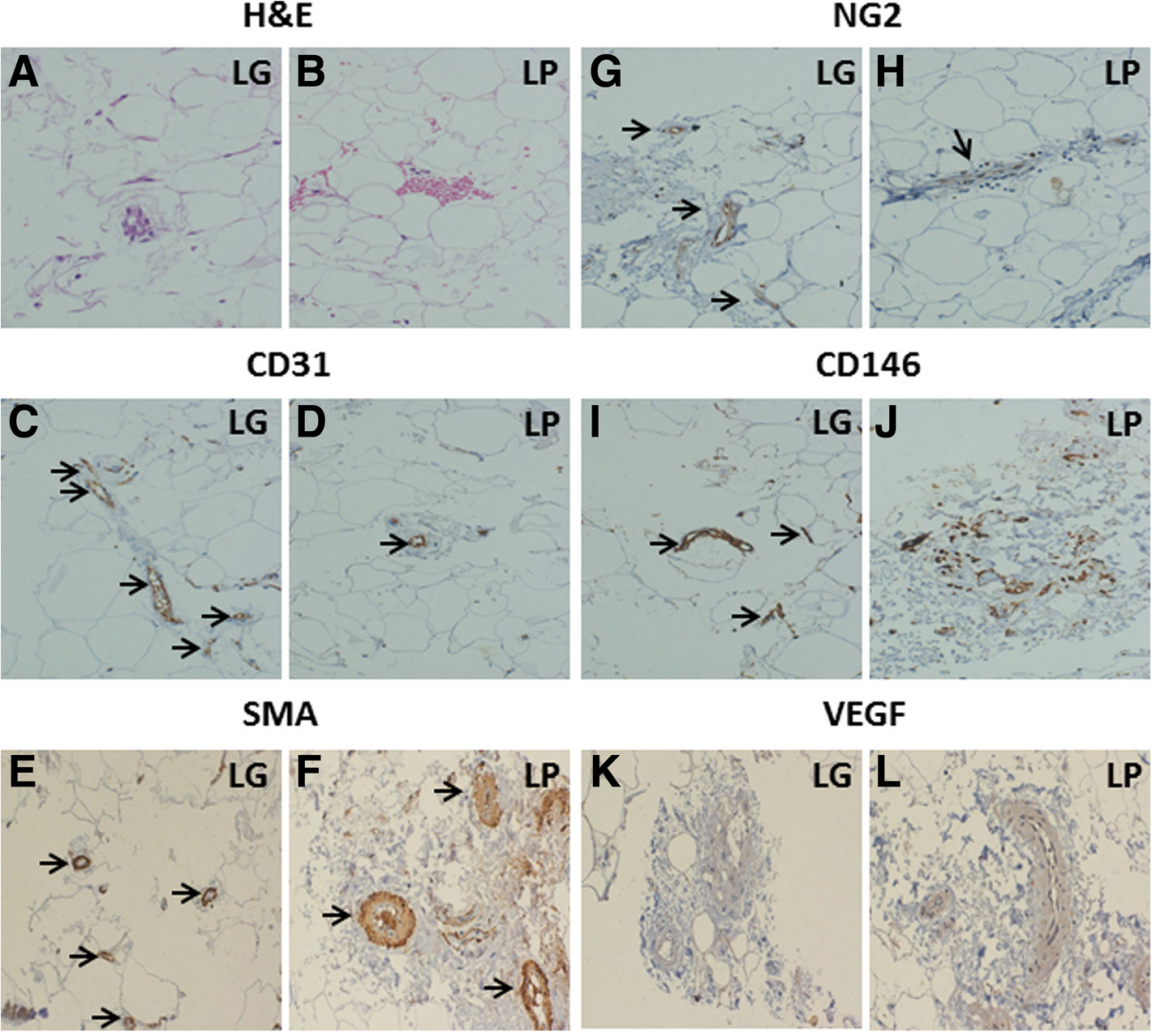
*Histological and immunohistochemical analysis of LG and LP*. H&E staining shows higher content of microvessels in LG (**a**) compared to LP (**b**). The immunohistochemistry shows an intense staining (black arrows) for the endothelial marker CD31 (**c** and **d**) and mural cell marker SMA (**e** and **f**). Staining with NG2 (**g** and **h**) was associated to microvessels and showed the presence of pericytes, that were more abundant in LG compared to LP (black arrows). Staining for CD146 (**i** and **j**), a marker present on both pericytes and endothelial cells, was more diffuse and intense in LP, while in LG was more associated to microvessels (black arrows). VEGF staining was present in the connective of both LG (**k**) and LP (**l**); LG showed less connective matrix than LP (Magnification 20x)

H&E staining and immunohistochemical analysis (Fig. 1) revealed that LG has a very high content of microvessels, compared to LP (Fig. 1a, b). Indeed, CD31^+^ cells are 10 times more in LG than in LP (Fig. 1c, d). SMA staining indicated that LP contains more larger vessels than LG (Fig. 1e, f). The analysis of NG2, a marker specifically expressed on pericytes but not on mECs [16], revealed that the number of pericytes, which are only present on microvessels [18], is higher in LG compared to LP (Fig. 1g, h). In contrast, CD146 seemed higher in LP, while in LG is more localized within the microvessels (Fig. 1i, j). The angiogenic factor VEGF is present in the connective of both LG and LP, despite LG showed less connective matrix than LP (Fig. 1k, l).

**Fig. 2:**
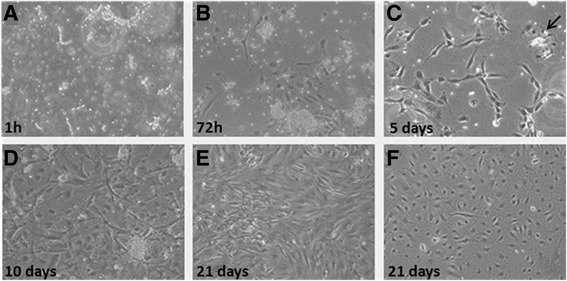
*Cultures of LG-MSCs upon collagenase dissociation*. Representative of five different Lipogems®-derived MSCs (LG-MSC) cultured from 1 h (**a**) to 21 days (**f**). Upon enzymatic digestion of human LG specimens, the obtained clusters were seeded in IMDM + 5 % FBS (**a**) in order to allow the adhesion and the proliferation of the released cells. After 72 h (**b**) all cultures showed the capacity to adhere on a plastic substrate and the typical stromal morphology. Starting from 5 days of culture (**c**) clusters of endothelial cells (black arrow) were highly distinguishable among the stromal cells and after 10 days (**d**) were removed from the culture by using CD31+ magnetic beads. Stromal and endothelial cell population was cultured separately and at 21 days reached complete confluence (**e**) and (**f**) respectively. (Magnification 10x)

**Fig. 3:**
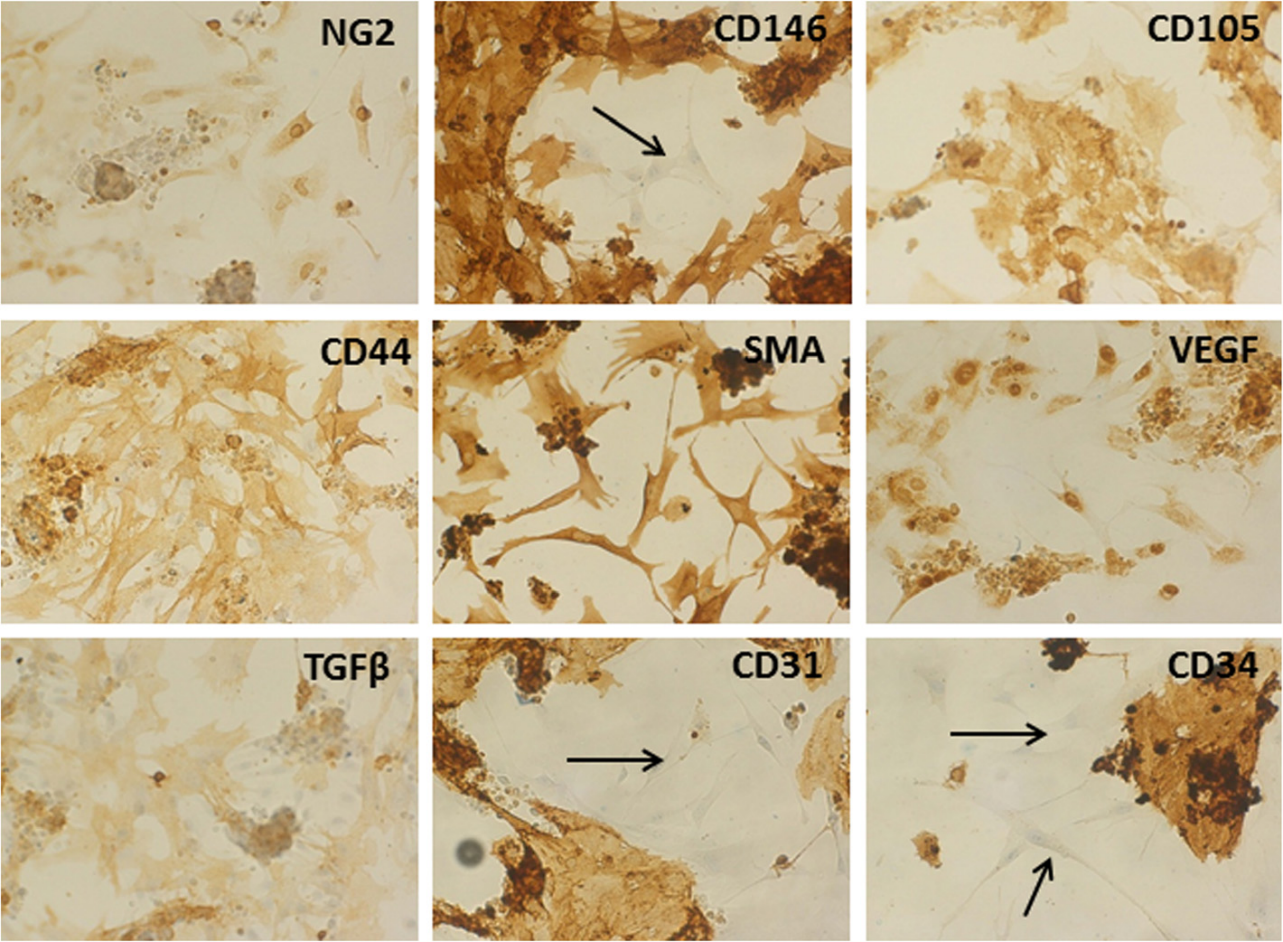
*Immunostaining of early culture of cells obtained after collagenase digestion of LG*. Endothelial, pericyte and mesenchymal markers were used to characterize the cells extracted from LG. Cells analyzed upon 48-72 h of culture, showed the presence of a significant number of cell colonies positive for CD31, CD34 endothelial markers, surrounded by fibroblastic negative cells (black arrows). Conversely, several fibroblastic-like cells were positive for the pericyte marker NG2, and the mesenchymal markers CD105, CD44 and SMA. Interestingly, CD146 stained very intensely both colonies and fibroblastic cells; very few cells were negative. Fibroblastic-like cells were also positive for TGFβ1 and VEGF, growth factors that are usually secreted by MSCs. (Magnification 20x)

**Fig. 4:**
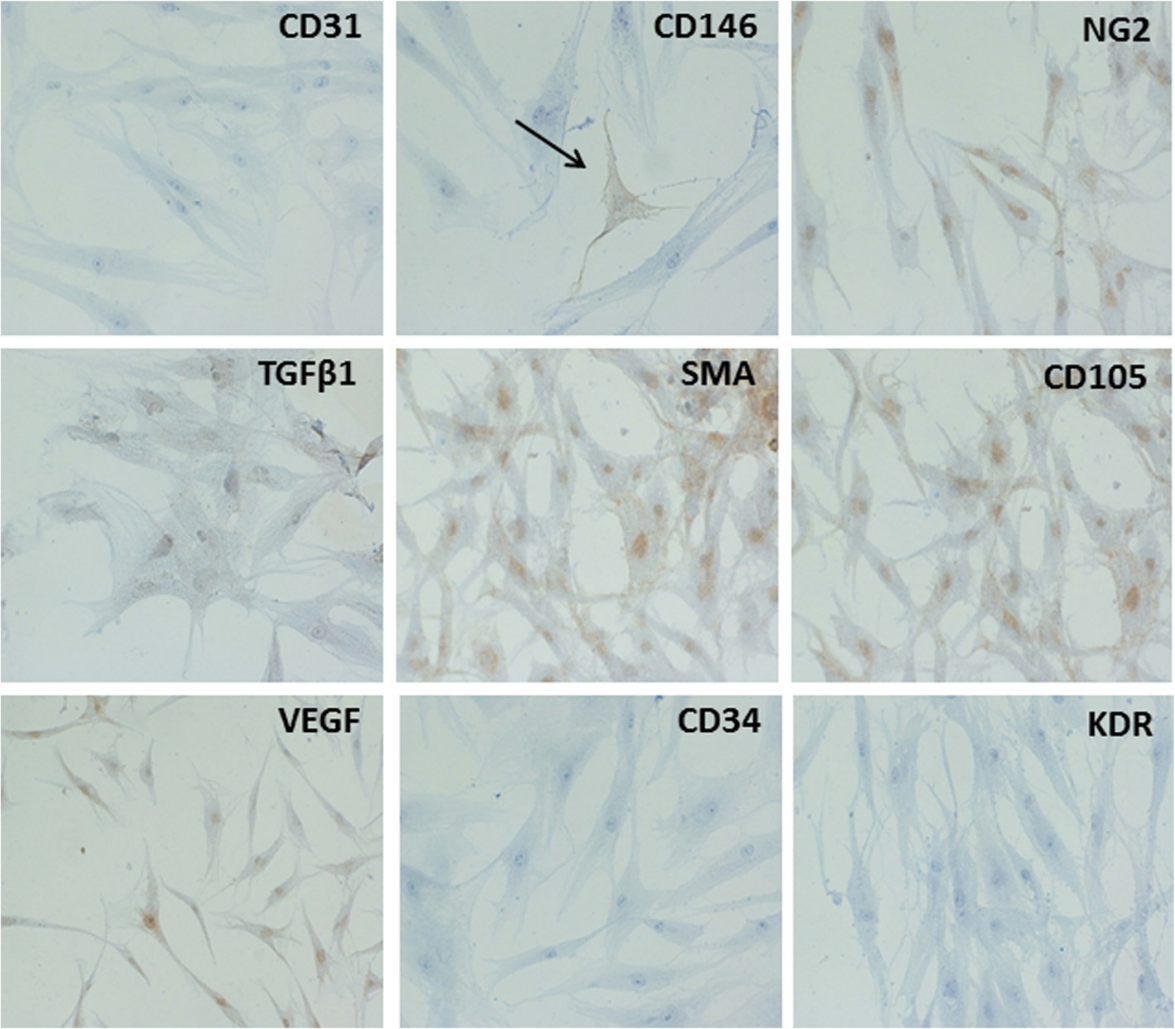
*Immunocytochemistry of secondary culture of LG-derived MSCs*. Immunocytochemistry was performed on LG-MSCs after 2–3 in vitro passages. Under these experimental conditions, LG-MSCs lost the expression of endothelial markers CD31, CD34 and KDR (a receptor for VEGF), while retaining pericyte and mesenchymal markers NG2, CD105 and SMA. Cells were still positive for TGFβ1 and VEGF. To note, CD146 positive cells (black arrow) were very few, suggesting that this marker in LG was probably mostly expressed by the cells of endothelial origin (Magnification 20x)

**Fig. 5:**
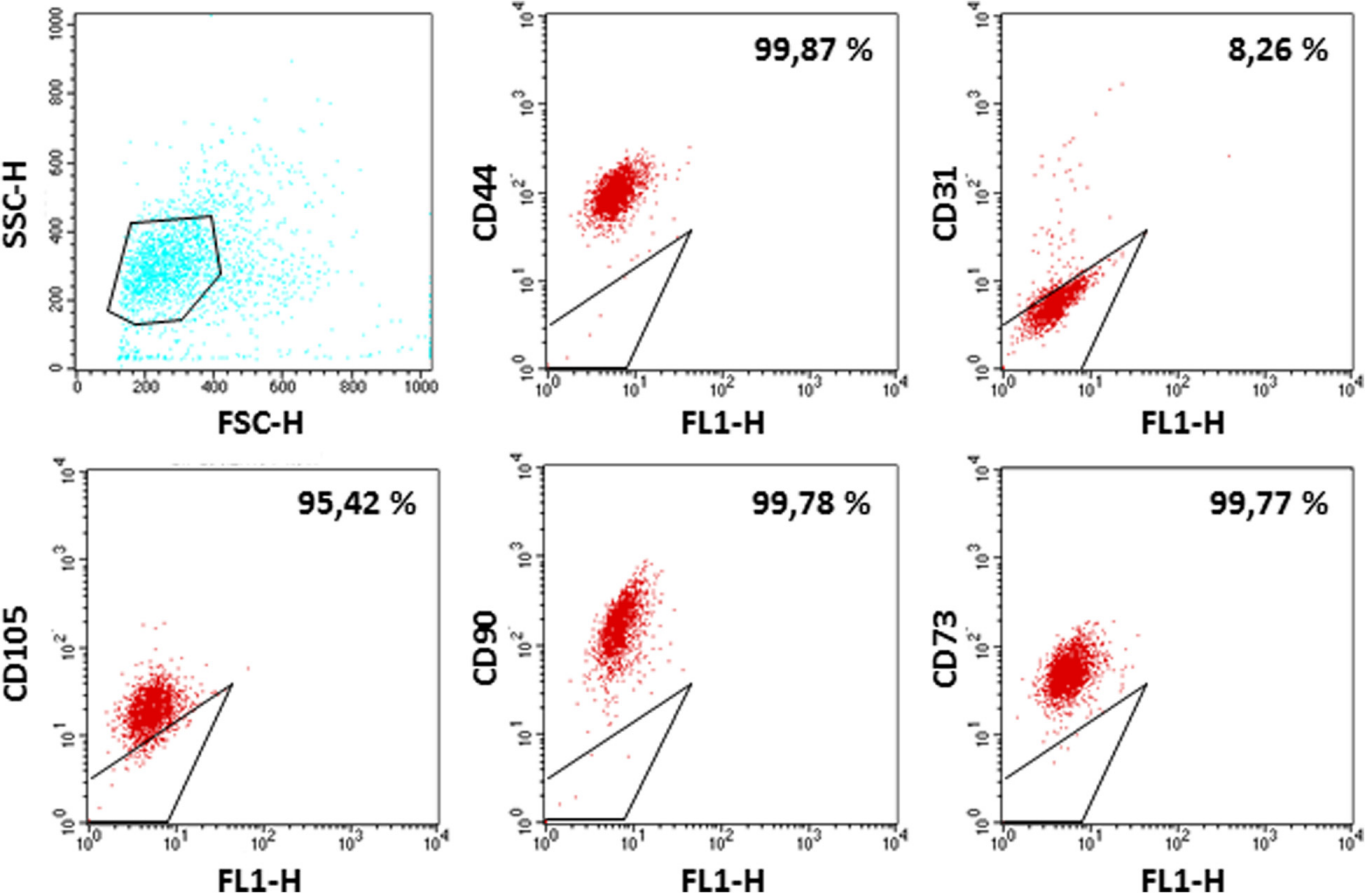
*Characterization of Lipogems®-derived MSCs*. The levels of CD44-PE, CD73-PE, CD90-PE, CD105-PE, CD31-PE markers in LG-MSCs were analyzed by flow cytometry. The percentage indicate the positivity of the cells for their relative surface markers, while the gate indicate the isotypic control (Magnification 10x)

To further investigate the cell content in LG specimens, collagenase treatment was performed. Upon enzymatic digestion of LG, several aggregates or cell clusters with capillary-like structures were observed (Fig. 2), as detected by light microscopy. Upon 48–72 h of culture, cell aggregates gave rise to colonies with endothelial-like morphology, surrounded by fibroblastic shape cells. During culture expansion, these fibroblastic shaped cells increased their number more rapidly than endothelial-like cells and, very often, grew above the endothelial-like colonies. By using magnetic beads coated with CD31, it was possible to separate the two different populations, one positive for CD31, with endothelial appearance, and, the second negative for CD31, with fibroblastic-like morphology (all data are summarized in Fig. 2). The immunostaining of early LG cell cultures (48-72 h) with endothelial, pericyte and mesenchymal markers indicated the presence of a significant number of cells positive for CD31, CD34 and CD146 (Fig. 3). Several cells were positive for NG2 CD44, CD105 and SMA. In addition, most of the cells were strongly positive for TGFβ1 and VEGF, usually secreted by MSCs. Interestingly, secondary cultures of LG-derived cells (2–3 in vitro passages), became negative for CD31, CD34 and KDR (a receptor for VEGF); CD146 was strongly reduced, whereas several mesenchymal and pericyte markers (CD105, CD44, SMA and NG2), as well as specific mesenchymal growth factors (TGFβ1 and VEGF) were maintained (Fig. 4). In summary, the immunostaining of early culture of LG-derived cells revealed the presence of two different populations: mECs and pericyte-associated MSCs. However, upon only 2–3 *in vitro* passages, cells with endothelial phenotype were lost and only cells with mesenchymal and pericyte features remained. The MSC phenotype of LG-MSCs cultures was also confirmed by FC analysis (cells were positive for CD90, CD73 and CD105 [12] - Fig. 5).

### LG and LG-MSC angiogenic properties

**Fig. 6:**
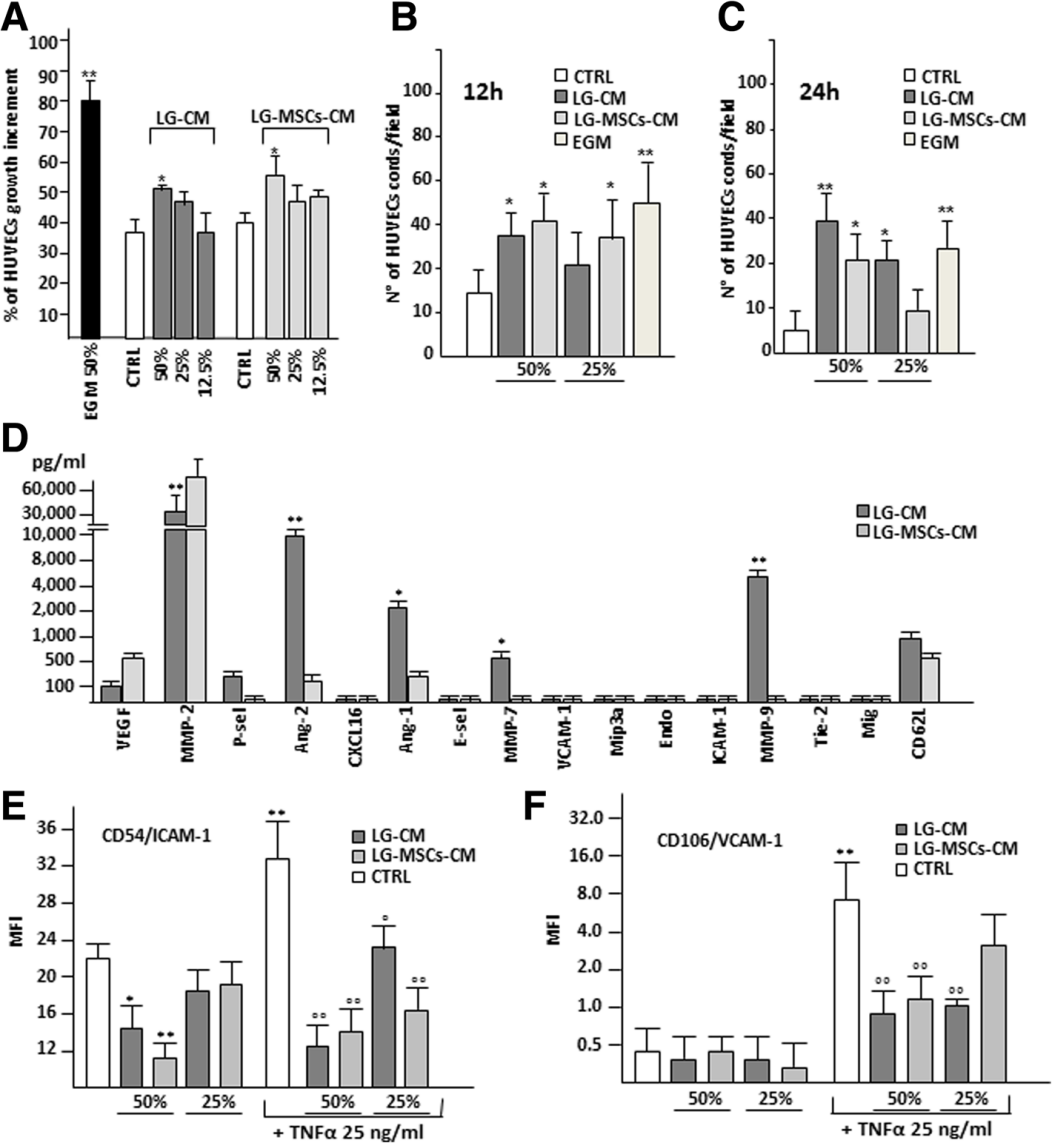
*Angiogenic properties of LG and LG-MSCs*. The angiogenic properties of LG and LG-MSCs were investigated by testing their CM. In (**a**) the effect of LG-CM and LG-MSCs-CM on HUVECs proliferation. EGM medium was used as positive control for proliferation, EGM medium without growth factors the negative control (CTRL) (* *p* < 0.05; ** *p* < 0.01 versus CTRL). In (**b**) the quantification of cord formations on MTG by HUVECs under the influence of LG-CM and LG-MSCs-CM at 12 h and at 24 h in (**c**) (* *p* < 0.05; ** *p* < 0.01 versus CTRL). In (**d**) the analysis of angiogenic molecules present in the secretome of LG-CM in comparison to LG-MSCs-CM. MMP2 was significantly reduced in LG-CM, while ANG-1 and ANG-2 were increased (* *p* < 0.05; ** *p* < 0.01 versus LG-MSCs-CM). In (**e**) and in (**f**) the effect of LG-CM and LG-MSCs-CM on the expression of HUVEC ICAM-1 and VCAM-1 respectively (* *p* < 0.05; ** *p* < 0.01 versus CTRL; °*p* < 0.05, °° *p* < 0.01 versus TNFα treatment)

It has been shown that LG-MSCs naturally express a set of vasculogenic genes and that LG, when transplanted into an experimental ischemic tissue, improved tissue repair possibly by stimulating angiogenesis [4, 5]. In order to broaden our knowledge, we here investigated whether LG and LG-MSCs were able to release molecules affecting angiogenesis. Collagenase digestion of LG was necessary to obtain a sufficient number of cells and to develop a culture in few days. Indeed, the mechanical dissociation, as well as the direct cultivation, required up to 3–4 weeks. The LG-CM and LG-MSCs-CM were then tested on HUVECs to evaluate proliferation, cord-like formation and ICAM-1 and VCAM-1 expression (Fig. 6). Under our experimental conditions, the addition of different dilutions of both LG-CM and LG-MSCs-CM improved only slightly HUVEC proliferation (Fig. 6a), but increased capillary-like tube formation after 12 h incubation (Fig. 6b) compared to control medium condition. However, at 24 h HUVEC cords spontaneously regressed under control medium condition, whereas in the presence of LG-CM a significant number of cords were still present. LG-MSCs-CM was less effective (Fig. 6c). These data suggest a higher capability of LG-CM in stabilizing neoformed vascular-like structures compared to their LG-MSCs counterpart.

It is well known that Angiopoietins (Ang-1 and Ang-2) are involved in vascular maturation [13]. Thus, we examined, together with a panel of other angiogenic-related molecules, LG and LG-MSCs secretome. Although the different procedures adopted to obtain LG-CM and LG-MSCs-CM cannot allow an absolute comparison, the Luminex assay seemed to indicate that LG secreted less VEGF and MMP-2, but a significant higher amount of Ang-1 and Ang-2 as well MMP-7 and MMP-9 compared to LG-MSCs (Fig. 6d). We next examined the capacity of LG-CM and LG-MSCs-CM to modulate HUVEC expression of ICAM-1 and VCAM-1. Different dilutions of LG-CM and LG-MSCs-CM were added to HUVECs cultured in the absence or in the presence of TNFα, an inflammatory cytokine (IC) able to enhance AM expression [26]. At 50 % dilution, the addition of either LG-CM or LG-MSCs-CM significantly reduced the expression of ICAM-1, both the base line as well as the increment induced by TNFα (Fig. 6e). VCAM-1 expression was significantly reduced by LG-CM; a similar trend was observed by adding LG-MSCs-CM only in the presence of TNFα (Fig. 6f).

### LG and LG-MSC anti-inflammatory properties

**Fig. 7:**
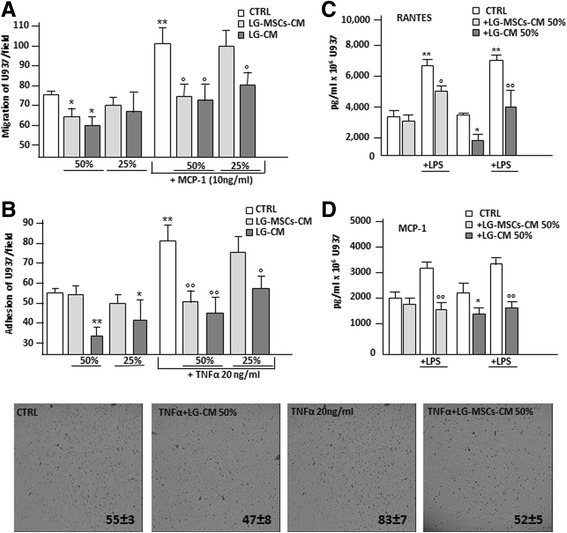
*LG-CM and LG-MSCs possess anti-inflammatory properties*. The capacity of LG-CM and LG-MSCs to affect inflammatory cell functions was tested on the U937 monocyte cell line. In (**a**) the effect of LG-CM and LG-MSCs-CM on U937 migration. Different dilutions of LG-CM and LG-MSCs-CM were placed in the lower compartment of a transwell insert (5um pore size). The chemokine MCP-1 at (10 ng/ml) was used as a positive stimuli for U937 migration. RPMI+ 0.2 % BSA was the control medium (CTRL) (* *p* < 0.05; ** *p* < 0.01 versus CTRL; °*p* < 0.05, versus MCP-1 treatment). In (**b**) adhesion of U937 to HUVECs monolayer in the presence of LG-CM and LG-MSCs-CM. TNFα at 20 ng/ml was used to activate HUVECs (* *p* < 0.05; ** *p* < 0.01 versus CTRL; °*p* < 0.05, °° *p* < 0.01 versus TNFα treatment). The photos below the figure show the morphological appearance of U937 attached to HUVECs primed with TNFα, LG-CM and LG-MSCs-CM after 30’ incubation (5x magnifications). In (**c**) and in (**d**) the release of RANTES and MCP-1 by U937 under the influence of LG-CM and LG-MSCs-CM stimulated or not with LPS (1ug/ml) respectively (*p < 0.05; **p < 0.01 versus CTRL; °*p* < 0.05, °° *p* < 0.01 versus LPS treatment)

It has been reported that MSCs are the guardians of inflammation [34, 37] and their injection ameliorates clinical signs in animals with induced inflammatory diseases [22]. We here investigated whether LG or LG-MSCs possess anti-inflammatory properties, in particular affecting some macrophage functions involved in inflammation (Fig. 7). U937, a human monocyte cell line, were used to investigate macrophage functions. We first examined the capability of LG-CM and LG-MSCs-CM of affecting U937 migration. Our results indicate that the presence of LG-CM and LG-MSCs-CM reduced U937 motility in a dose dependent manner (Fig. 7a). The inhibitory effect of LG-CM and LG-MSCs-CM on U937 was more evident when the migration test was performed in the presence of MCP-1/CCL-2, a chemokine shown to recruit inflammatory cells to the site of inflammation [40]. Under this experimental condition, both LG-CM LG-MSCs-CM were effective, particularly at higher dilutions (Fig. 7a). Because the adhesion of inflammatory cells on the vascular endothelium is a hallmark of the inflammatory process [20], we next analyzed whether LG-CM and LG-MSCs-CM affect U937 adhesion on a HUVEC monolayer in the absence or in the presence of TNFα, an inflammatory cytokine known to enhance the stickiness of inflammatory cells on the endothelium [26]. The addition of LG-CM (1:2 and 1:4 dilutions) significantly inhibited U937 adhesion both in the absence and in presence of TNFα. LG-MSCs-CM, reduced the adhesion on HUVECs only following TNFα stimulation (Fig. 7b). Finally, we analyzed the ability of U937 to secrete RANTES and MCP-1, two chemokines involved in inflammation [25]. The incubation of U937 with LG-CM and LG-MSCs-CM at 1:2 dilution significantly inhibited RANTES secretion both under basal condition and upon stimulation with LPS (Fig. 7c). Similar results were obtained for MCP-1 (Fig. 7d).

## Discussion

Because of their strong functional and phenotypic similarities with bone marrow MSCs [21] and their easy isolation through a minimally invasive surgery, Ad-MSCs are considered an attractive biological tool for advanced cell therapies in humans [8]. In general, the clinical use of MSCs is strongly limited because of the significant manipulations required during isolation procedures and *in vitro* expansion, thus falling under the highly demanding requirements imposed by cGMP guidelines. To overcome this problem, new approaches using minimal manipulation on human cells, tissues and tissue-based products have been developed. Recently, prof. Tremolada and colleagues developed and patented an innovative technique to process fat specimens, such as LP, without the use of enzymes, to obtain an autologous micro-fragmented adipose tissue product ready to be injected into patients or eventually cryopreserved for future applications [4]. Application of LG in humans [35, 38, 39] as well as in experimental animal ischemic disease models have shown some beneficial effects possibly mediated by the capability of LG to release vasculogenic/angiogenic molecules [5]. In an attempt to better understand the functional properties of LG, we here investigated its angiogenic and anti-inflammatory features *in vitro*. To this end, fresh LG preparations were cultured in serum free condition to better analyze their secreted factors. In parallel, from the same amount of LG we extracted and cultured LG-MSCs to determine if a similar functional activity was retained. Comparative activity between LG and their isolated cellular content i.e. LG-MSCs was not the object of this study because of the impossibility to estimate the number of cells present in LG preparations and the different culture conditions.

At the histological level, LG is very rich in microvessels and this was confirmed by the intense positivity of LG specimens for CD31 and SMA (endothelial and mural cell markers). In addition, the wall of the microvessels in LG was also positive for CD146 which is considered a pericyte marker, but it is also known to be expressed on ECs particularly under inflammatory conditions [2].

Differently from other previous studies that investigated the expression of CD146 and CD34 as pericyte and mEC markers respectively [4], we here analyzed the expression of NG2 as univocal marker for pericytes [27]. We found that NG2 positive cells were significantly present in LG microvessel, and the results were also confirmed by the immunocytochemical analysis of the freshly isolated cells derived from LG upon collagenase digestion. Differently from other published results [4, 9], the use of the enzyme was necessary to obtain a sufficient number of cells in 7–14 days, while generally 4–6 weeks are required for a direct place of LG in culture medium or a mechanical dissociation using a Pasteur pipette. Substantially, we observed that LG was composed by an abundant number of mECs islets (positive for CD31, CD34 and CD146) surrounded by several stromal cells expressing the typical mesenchymal markers (CD44, CD105, TGFβ1), most of them positive for NG2. In summary, our results broaden previous data showing, for the first time, that most of LG-MSCs are of pericyte origin and positive for NG2. Because the pericytes within the microvessels have been proposed to be the real immature precursors of all different tissue-derived MSCs [7, 10], we can conclude that immature MSC progenitors are highly concentrated in LG. This conclusion is also supported by the analysis of the secondary culture of LG-MSCs, where, besides a high positivity for the typical MSCs markers CD90, CD105, CD73 [12] most of the cells were also positive for NG2 (almost 100 %). Interestingly, CD146 was significantly reduced in the secondary culture, whereas mECs were almost absent. Thus, at least within the adipose tissue, CD146 stains mostly cells of endothelial origin rather than pericytes.

The analysis of LG angiogenic activity demonstrated that it stimulates only marginally HUVEC proliferation, whereas it significantly reduces AM expression (ICAM-1 and VCAM-1) and improves cord-like formations, indicating a preferential ability of LG-CM to favor vascular stability and maturation. This result was partially confirmed by the analysis of its secretome that indicated a high content of both Ang-1 and Ang-2, and low levels of VEGF and MMP2 compared to LG-MSCs-CM that, in this context, had an effect on HUVECs but with an apparent lower efficacy compared to LG-CM, although a comparative analysis between the two CMs cannot be done. In line with our previous observation on Ad-MSCs isolated through enzymatic digestion [6], both LG-CM and LG-MSCs-CM did not contain inflammatory cytokines. We also found for the first time that both LG-CM and LG-MSCs-CM have potent anti-inflammatory properties. Indeed, they both reduced migration, adhesion to an activated EC monolayer and release of RANTES and MCP-1 chemokines of U937, monocytes of tumorigenic origin [23] used as a valid model to investigate the inflammatory properties of monocytes [23, 36]. Taken together our results clearly indicate that LG and its purified MSCs content are able to block several important monocyte inflammatory functions.

## Conclusion

Our results point at LG as a valid micro-fragmented fat derivative that retains anti-inflammatory and vascular stability properties even higher than its purified and cultured Ad-MSC content. Because of the minimal manipulation, LG bypasses the complex requirements of GMPc guidelines, with a dramatic reduction of the costs for cell-based therapies on human patients. The major limitation is represented by the technical difficulties in the estimation of the real cell content and, in particular, the number of MSCs/ml of LG. In addition, the impossibility at this moment of being inoculated in patients through the systemic route (intravenous injection) limits its clinical use at a local treatment. Finally, because LG has an impressive secretory activity, its CM may be used not only for the identification of active molecules involved in angiogenesis or inflammatory processes, but also to produce microvescicles [14] that might be used for alternative therapies of inflammatory diseases in human.
